# Appraisal of testicular volumes: volumes matching ultrasound values referenced to stages of genital development

**DOI:** 10.1186/s13633-017-0046-x

**Published:** 2017-07-17

**Authors:** Juan F. Sotos, Naomi J. Tokar

**Affiliations:** 1 Department of Pediatric, College of Medicine, The Ohio State University, Nationwide Children’s Hospital, Section of Pediatric Endocrinology, 700 Children’s Drive, Columbus, OH 43205 USA; 2 Nationwide Children’s Hospital, Section of Pediatric Endocrinology, 700 Children’s Drive, Columbus, OH 43205 USA

**Keywords:** Testicular volumes, Genital development in males, Genital stages, Orchidometers

## Abstract

**Background:**

Testicular volumes obtained with orchidometers or external linear measurements in the scrotum (centimeter ruler or calipers) grossly over-estimate ultrasound volumes, have much variability and may not be accurate or reproducible. The reference of the values obtained by orchidometers or US, to age or Tanner stages is not useful to determine the normal values for stages of puberty, because overlapping of ages and values. Pubertal development is determined by two events, genital and pubic hair development, that should be analyzed independently because one could be out of step with the other. The ultrasound (US) measurement of testicular volumes is the gold standard but is somewhat inconvenient, because it requires another procedure and, mainly, is costly.

The solution of the problems would be to determine testicular volumes matching US values, from the width of the testis obtained in the scrotum with a centimeter ruler, by formulas recently described, and to reference them to the stages of genital development.

**Methods:**

The width and length of the testes in the scrotum with a centimeter ruler were obtained in 159 study subjects, in different stages of genital development and adults, for a total of 318 testicular determinations, from the age of 3 to 34 years. The width obtained in the scrotum was corrected by subtracting the values of the double scrotal skin (ss). The formulas were then applied and the testicular volumes matching US values were calculated. The volumes and the range of ages for different stages of genital development were determined. Penile measurements were obtained in 145 subjects and pubic and other hair recorded.

Paired and unpaired 2 tail student t-test was used to compare the means of the different groups expressed as means and SD and, in addition the Wilcoxon rank sum test and Bootstrap methods for the testicular volume groups. A *p* value of 0.05 or less was considered significant.

The Institutional Review Board (IRB) of Nationwide Children’s Hospital determined that this study did not require IRB approval.

**Results:**

With a simple measurement of the width of the testis in the scrotum, with a centimeter ruler, testicular volumes matching US values were calculated and normative values for each stage of genital development were determined.

**Conclusion:**

This information should solve present problems.

**Electronic supplementary material:**

The online version of this article (doi:10.1186/s13633-017-0046-x) contains supplementary material, which is available to authorized users.

## Background

The determination of the testicular volume is of considerable importance to assess the onset, progression and disorders of puberty, including the effect of cryptorchidism and orchiopexy, hypogonadism with respect to tubular function, the effect of a varicocele, abnormal testicular development, damage to the testis by torsion or inflammation, compensatory hypertrophy, detection of Klinefelter syndrome, effect of the administration of sexual steroids or drugs, and, in adults, assessment of fertility. Low testicular volume correlates with tubular size, function and spermatogenesis [1].

In addition, the testicular volume is of interest to assess macroorchidism, such as in Fragile X syndrome, FSH secreting pituitary macroadenomas, immunoglobulin superfamily member 1 (IGSF1) deficiency syndrome [2], long-standing hypothyroidism, adrenal rest cell tumors in congenital adrenal hyperplasia, lymphomas and so on.

A number of clinical methods have been used for the measurement of testicular volumes in the scrotum. Some use an ordinary ruler or sliding calipers [3–5], others use orchidometers [6–10]. Testicular volume is usually measured using the Prader orchidometer. All the clinical methods calculate the volumes by the ellipsoid equation Width^2^ x Length x $\frac{\uppi\ }{6}$ (W^2^ x L × 0.52), and overestimate US volumes.

Ultrasound measurements have a high degree of accuracy and reproducibility and are the standard for quantitation of testicular volume [11, 12].

The volumes obtained by ultrasound have been calculated by different ellipsoid equations. Some have used only the width (W) and length (L) of the testes, W^2^ x L x $\frac{\uppi\ }{6}$that when resolved is W^2^x L × 0.52 = Volume. More frequently they have included the height (H), W x H x L × 0.52 and others, recently, have used the constant 0.71 (suggested by Lambert [13]), to closely match the “true” testicular volumes obtained by water displacement, W x H x L × 0.71 = Volume [14, 15].

Formulas, equivalent to the ellipsoid equations used, with inclusion of the values observed in ultrasound measurements, were developed to approximate or match ultrasound volumes, with corrections of the width and length of the testis obtained in the scrotum, to avoid the inclusion of the scrotal skin (ss) and epididymis; the Width minus the double scrotal skin (W-ss), to match the US width.

For the US equation W x H x L × 0.52, the equivalent formula would be (W-ss)^3^ × 0.64.

**Fig. 1:**
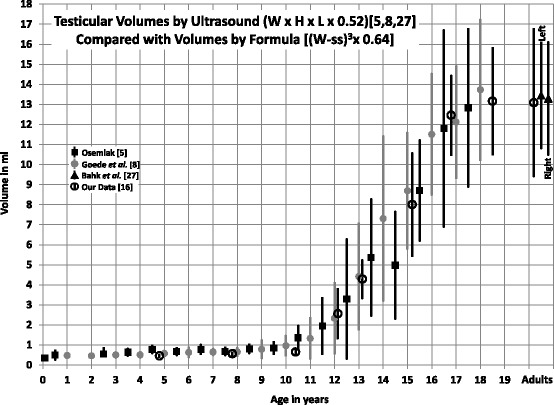
Testicular volumes (Mean ± 1 SD) obtained by ultrasound in normal children and adults reported by 3 groups compared with volumes in our subjects obtained clinically by formula [(W-ss)^3^ × 0.64] [16, 27]

If the constant 0.71 instead of 0.52 is used, then the US equation would be W x H x L × 0.71, and the equivalent formula (W-ss)^3^ × 0.88. These formulas were validated and values matching US values reported - Fig. 1. (For further details on equations and formulas, see additional file and reference [16]).

### There are Problems

1. The testicular volumes obtained with orchidometers grossly overestimate ultrasound values, mainly because the inclusion of the scrotal skin and epididymis, have much variability and may not be accurate or reproducible; this has been amply documented [9–12, 17, 18]. The ultrasound measurement, however, is somewhat inconvenient because it requires another procedure and, mainly, it is costly. It does not appear practical or reasonable to use ultrasound to assess the onset and progression of puberty or to assess some of the other conditions that have been mentioned.
2. The reference of the values obtained by orchidometers or ultrasound to age or Tanner stages is not useful to determine the normal values for stages of puberty, because overlapping of ages and values.

### Solutions

Pubertal development is determined by two events: pubic hair (from adrenarche and gonadarche) and genital development (testes, scrotum and penis from gonadarche). Because one could be out of step with the other, Tanner [19] recommended for genital and pubic hair development to be analyzed independently.

1. Formulas equivalent to the ellipsoid equations were developed that, from the width of the testis obtained in the scrotum with a centimeter (cm) ruler, can yield testicular volumes matching ultrasound volumes. That should solve the problem of overestimation with orchidometers.
2. Reference of the volumes so obtained to stages of genital development (testes, scrotum and penis only), should solve the second problem of reference to age or Tanner stages.
  The aim of this communication is to conduct an analysis of the problems, and to report normative values for testicular volumes matching ultrasound values for different stages of genital development and adults.

## Methods

The US observations in our hospital of the width, height, length, height/width and length/width ratios and volumes of 110 testes, from 55 children from 1 month to 17 years of age, were reviewed.

The width and length of the testis in the scrotum, with a cm ruler, were obtained in 159 study subjects in different stages of genital development and adults for a total of 318 testicular determinations, from the age of 3 to 34 years; a cross-sectional study. The width of the testis obtained in the scrotum was corrected by subtracting the values of the double scrotal skin. The formulas were then applied and the testicular volumes matching ultrasound values were calculated. The volumes and the range of ages for different stages of genital development were determined. The 159 study subjects consisted of 42 normal and 117 patients attending the endocrine clinic who had normal growth and gonadal development. Penile measurements of the length, while gently stretched, from the pubopenile skin to the tip of the glans, and of the width, at the mid-shaft, after smoothing the skin were obtained in 145 subjects. Pubic and other hair (axillary, inner thigh, linea alba, abdominal, chest and facial) were recorded.

The Institutional Review Board (IRB) of Nationwide Children’s Hospital determined that this study is a retrospective record review for quality improvement in accuracy, diagnosis and quality of care for appraisal of testicular volumes, and does not meet the definition of human subjects research under 45 CFR part 46, and consequently, this study does not require IRB approval.

### Statistical analysis

Paired and unpaired 2-tail student t-test was used to compare the means of the different groups expressed as means and standard deviations (SD).

The testicular volumes of genital stage (G) groups and Adults had non-normal distribution. Data transformation to normality was not possible, because their distribution varied by group. So, the data is presented as the median and interquartile range, instead of the mean and standard deviation. Pairwise group comparisons of adjacent G groups were run using three methods: the Wilcoxon rank sum test [20] and the Bootstrap method [21] that do not assume the data have normal distribution, and the two-sample Student t-test, because although the data deviate from normality, the group sizes (between 22 and 100) may be large enough to tolerate some degree of non-normality. A *p* value of 0.05 or less was considered significant.

All of the three tests for G1 vs G2, 2 vs. 3, 3 vs. 4, and 4 vs. 5 showed a highly significant difference for these pairs of groups, *p* < 0.00001. There was not difference between G5 and Adults *p* > 0.05, <0.1.

All analyses were run in SAS 9.4 (Cary, NC).

### Results-analysis of the problems

**Fig. 2:**
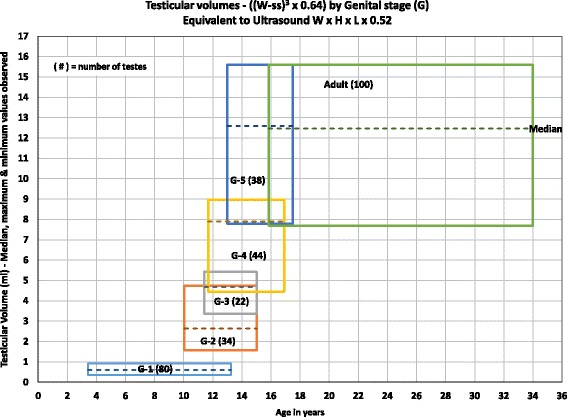
Testicular volumes ((W-ss)^3^ × 0.64) for stages of genital development (G), equivalent to ultrasound W x H x L × 0.52. The boxes for the testicular volumes of each stage of genital development and adults were determined as indicated in the methods. The width of the testis in the scrotum was measured with a centimeter ruler. The stages of genital development and range of ages were identified and the volumes were calculated by the formula (W-ss)^3^ × 0.64. The minimal and maximal values observed (not standard deviations) in ml were plotted in the ordinance and the range of ages in the abscissa. The boxes and the figure will be the same for volumes calculated with formula (W-ss)^3^ × 0.88 or equation W^2^x L × 0.52 (in Additional file 1: Figure S2 & Figure S3) but with different numbers for the minimal and maximal volumes

1. Overestimation: A number of orchidometers have been described; the Prader orchidometer, described in 1966 [6] and the Takihara orchidometer (also known as Rochester orchidometer) [10] described in 1983 are probably the most frequently used. We conducted an analysis of this overestimation. The ratios of the volumes obtained by the Prader orchidometer and by ultrasound by Goede et al. [8], Table 1, were 1.8 folds to 2 for adults, 2 to 2.5 for pubertal and up to 3.4 folds for prepubertal males, and similar ratios by the caliper external measurements by Osemlak et al. [5]. The greater overestimation for prepubertal than for adults is related to the greater proportion of scrotal skin over width of the testis: ($\frac{ss}{W}$) for prepubertal males = 15%, for pubertal = 8.6%, and adults = 7.2%. Moreover, since the scrotal measurements do not include height (W^2^ x L × 0.52), there would be an overestimation of the US volumes (W x H x L × 0.52) of about 20% to 30%. Height often is 0.7 to 0.8 of the width.
  The testicular volumes we obtained by the measurement of the width and length of the testis in the scrotum with a cm ruler (W^2^ x L × 0.52) are similar to those obtained by the Prader orchidometer (Additional file 1: Figure S1). The ratio of the volumes we obtained with a cm ruler in the scrotum and the ultrasound values by our formula (W-ss)^3^ × 0.64 (equivalent to ultrasound (W x H x L × 0.52)) were 1.8 folds for adults, 1.6 to 2 for pubertal, and 1.9 to 3.5 folds for prepubertal males, similar to those obtained with the Prader orchidometer by Goede et al. [8]:
2. Reference of values: Another problem is the reference of values obtained by orchidometers or ultrasound to age. Because of the overlapping of ages for the development of different stages of genital stages, the reference by age alone does not provide normative values for different stages of genital development and may not detect microorchidism, macroorchidism, early or delayed development etc. Furthermore, because of the overlapping, the range of values for any year of pubertal age, interpreted as normal, is very wide. The volumes we observed for different stages of genital development, by our formula (W-ss)^3^ × 64 are shown in Fig. 2. The boxes for each stage of genital development and for adults show the minimal and maximal values of the volumes in the ordinance and the age range in the abscissa. A normal 14-year-old could have a genital development (G) of stage 2, 3, 4, or 5 and the normal volumes could be from 1.5 to 15.6 ml. If a 14-year-old has a volume of 6 ml, that will be judged to be normal, but actually will be high (macroorchidism?) if he has a genital development of G2 or G3 or low (microorchidism?) if he has a G5. Similar statements are applicable to other of our ultrasound values ((W-ss)^3^ × 0.88) or linear measurements (Additional file 1: Figure S2 and S3). The comments are also applicable to the values referenced by age recently reported [22] providing normalized-smoothed values by US and Prader Orchidometer in standard deviations. The normal values reported for testicular volumes of a 14-year-old by ultrasonography (W x H x L × 52) are 1.3 ml (−2 SD) to 14.5 ml (+2 SD) similar to ours with the formula (W-ss)^3^ × 0.64. For similarity of other values see Additional file 1: Table S1. Thus, the testicular volume cannot be referred by age only, but also by stage of genital development.
  There are also problems with the reference of the values obtained by ultrasonography or orchidometers to Tanner stage. The staging system most frequently used is known as Tanner stages consisting of genital and pubic hair changes [23]. The lack of pubic hair indicates Tanner stage 1 or prepubertal. Pubic hair may not be present, normally, until the age of 13 or 13 ½ or even 15 years in some normal subjects. By that time some of the subjects may have genital development of stage 2 or 3. Actually, Marshall and Tanner in 1970 [24] reported genital development of stage 3 and 4 in males on Tanner stage 1. So, it would be a problem and confusing to reference a boy with stage 3 of genital development (pubertal) without pubic hair, as Tanner Stage 1 (prepubertal). That is the reason Tanner recommended to analyze the genital development and pubic hair development independently, because one could be out of step with the other. Some of our subjects with Tanner stage 1 were pubertal (G2 & G3), (Table 2). Joustra SD et al., [22] obtained testicular volumes, by ultrasonography of up to 10 ml, when the normal values are less than 1. Obviously, there was gonadal development without pubic hair.

**Table 1:** Comparison of testicular volumes by prader orchidometer [8] & by caliper [5] to ultrasound measurements

Age (years)	Prader mean (ml)	US mean (ml)	Ratio	Caliper mean (ml)	US mean (ml)	Ratio
1	1.64	0.48	3.42	1.52	0.53	2.87
2	1.57	0.46	3.41	1.54	0.53	2.91
3	1.57	0.51	3.08	1.48	0.64	2.31
4	1.74	0.51	3.41	1.86	0.77	2.42
5	1.83	0.58	3.16	1.54	0.66	2.33
6	1.94	0.63	3.08	1.76	0.76	2.32
7	2.03	0.65	3.12	1.74	0.67	2.60
8	2.08	0.66	3.15	1.90	0.80	2.38
9	2.31	0.79	2.92	2.37	0.84	2.82
10	2.67	0.97	2.75	2.52	1.30	1.94
11	3.48	1.33	2.62	3.08	1.70	1.81
12	5.73	2.33	2.46	5.79	2.57	2.25
13	10.16	4.42	2.30	8.65	4.71	1.84
14	15.00	7.31	2.05	10.02	4.53	2.21
15	19.04	8.69	2.19	15.78	8.48	1.86
16	23.92	11.51	2.08	19.29	11.14	1.73
17	24.63	12.12	2.03	20.60	12.42	1.66
18	24.41	13.73	1.78

**Table 2:** Findings for stages of genital development clinically testicular volumes by (W-ss)^3^ × 0.88*

Genital stage	Age range (years)	Testis width in scrotum (cm)	Testicular volume observed (ml)	Length of penis (cm) mean ± SD observed range	Hair stages	
					Pubic hair	Other hair
1	Up to 13.25	**<1.3**	**<1.27**	**Early childhood 4.8 ± 0.64 Observed 3.5 to 6.0**	**None**	
2	10 to 15	**>1.5 to 2.1** ^**#**^	**>2.1 to 6.5** ^**#**^	**Early childhood 5.3 ± 0.75 Observed 4.0 to 6.5**	**None or sparse growth of downy or curled at the base of penis**	Early pubic hair is usually from adrenarche
3	11.4 to 15	>2.0 to 2.2^#^	4.6 to 7.4^**#**^	**Growth of penis 7.4 ± 0.49 Observed 7.0 to 8.0**	**No hair to sparse growth of darker and curlier**	Pubic & axillary hair from adrenarche & gonadarche
4	11.6 to 17	>2.1 to 2.6^**#**^	6 to 12.3^**#**^	**Development of glans 8.6 ± 1.29 Observed 6.5 to 12.0 cm**	**Abundant but not filling whole pubic area**	As above, Not in inner thigh
5	13 to 17.5	**>2.5 to 3.1** ^**#**^	10.7 to 21.4^**#**^	9.5 ± 0.99 Observed 7.5 to 11.0	**Adult, abundant, inverse triangle**	**Inner thigh No in lineal alba**
Adult	> 16	>2.5 to 3.1	10.6 to 21.2	9.7 ± 1.01 Observed 8.0 to 11.0	**Abundant**	**Linea alba abdomen or chest or beard from gonadarche**

For testicular volumes calculated by W x H x L × 0.52, divide Values reported by 1.365 (0.71/0.52 = 1.365). The findings were obtained as indicated in the methods. The width of the testis was measured. The stage of genital development (testes, scrotum and penis only) as defined by Tanner, and range of years were identified. Testicular volumes were calculated. Penile length was measured. Pubic and other hair observed was recorded, but not included in determining stages 1 to 5 of genital development

**#** = *p* < 0.0001

* Testicular volumes obtained by (W-ss)^3^ × 0.88, equivalent to ultrasound volumes calculated by W x H x L × 0.71

Most helpful findings are **bolded**

### Solutions of the problems

1. To avoid the overestimation of ultrasound testicular volumes by orchidometer, one can use the simple measurement of the width of the testis in the scrotum with a cm ruler, and apply the formulas equivalent to the ellipsoid equations that would yield testicular volumes matching ultrasound volumes: the formula (W-ss)^3^ × 0.64, equivalent to the ultrasound equation W x H x L × 0.52, that is still used for some providers, or the preferred one, presently, formula (W-ss)^3^ × 0.88, equivalent to the ultrasound equation W x H x L × 0.71 (see later).
  Correlation of the volumes obtained for the different stages of genital development will provide normative data and solve the problem of reference to age and Tanner stages.

#### Stages of genital development

The main characteristics of the stages of genital development (testes, scrotum, and penis) separate from pubic hair development were well defined by Tanner by photography [19] (Additional file 1: Figure S4). There is no mention of pubic hair in genital stages and all these changes are related to gonadal activity. The stages of pubic hair development were also well defined [19]. Table 2 shows our findings for different genital stages. We measured the width of testis in the scrotum, calculated the volumes, and measured the penile length, for each stage of genital development and for adults. Our findings for different G-stages have not previously been reported, to our knowledge. The pubic and other hair were recorded. Table 2 could be quite helpful for the identification of the stage of genital development. Let’s consider a 14-year-old who has some enlargement of the testes, but still an infantile penis and no pubic hair. He would be a normal Stage 2 of genital development beginning puberty with enlargement of the testes, by development of seminiferous tubules from the effect of FSH, but an infantile penis because he does not have yet LH stimulation of Ledig cells and testosterone secretion. Pulsatile secretion of LH comes normally about 6 months or later from the pulsatile secretion of FSH. He does not have pubic hair because he does not have yet adrenarche or testosterone from gonadarche. If a different 14-year-old has further enlargement of the testes, and growth of the penis in length and width from testosterone, he would be stage 3. He may have some or no pubic hair (the effect of testosterone is seen sooner in the penis than in pubic hair). If another 14 or 15-year-old has further growth of the testes and penis and development of the glans, he would be Stage 4. The pubic hair would be from adrenarche and gonadarche.

The values of the penile measurement are included in Table 3. The difference between the means of the penile length for G3 and G2 is highly significant (*p* < 0.001). Even though these stages are supported by serum levels of FSH, LH, and testosterone, one does not need to obtain them.

**Table 3:** Penile Length & Width

Genital stage	Age (years) range	Number	Length (cm) mean ± SD	Width (cm) mean ± SD
G-1a	3.4 to 6.5	10	4.77 ± 0.68	1.46 ± 0.18
G-1b	7.2 to 8.7	10	4.80 ± 0.56	1.40 ± 0.20
G-1c	8.9 to 13.2	16	4.88 ± 0.67	1.66 ± 0.20
G-1 all	3.4 to 13.2	36	4.83 ± 0.64	1.53 ± 0.23
G-2	10.0 to 15.0	16	5.28 ± 0.74*	1.74 ± 0.25
G-3	11.4 to 15.0	10	7.40 ± 0.49**	2.24 ± 0.37
G-4	11.7 to 16.9	21	8.57 ± 1.29*	2.77 ± 0.36
G-5	13.0 to 17.5	19	9.47 ± 0.99*	3.22 ± 0.28
Adult	15.8 to 34.0	43	9.66 ± 1.00	3.09 ± 0.39

Difference between the means: *p < 0.05; **p < 0.001

G-1a, b, c: The only criterion for separation is age

**Fig. 3:**
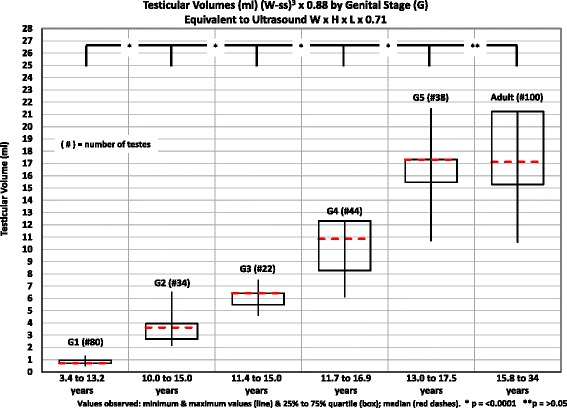
Testicular volumes ((W-ss)^3^ × 0.88) for stages of genital development (G), equivalent to ultrasound W x H x L × 0.71

**Fig. 4:**
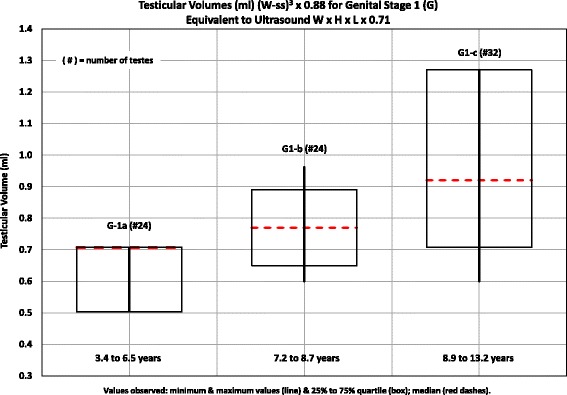
Testicular Volumes ((W-ss)^3^ × 0.88) for genital stage 1 (G), equivalent to Ultrasound W x H x L × 0.71. Age is the only criterion for the separation of G1a, b, and c. There is some growth

The testicular volumes obtained in our subjects for each stage of genital development and adults are included in Table 4. The volumes were calculated with the formula (W-ss)^3^ × 0.88, because the use of the 0.71 constant, presently, is the preferred one, because it closely matches the “true” testicular volumes obtained by water displacement (W x H x L × 0.71). The range of ages is consistent with that reported by a number of authors [24, 25]. To facilitate the provider to compare the volumes he/she obtains with the normal volumes obtained by us, the median, quartiles, and minimal and maximal values we observed are included in Figs. 3 and 4. The testicular volumes for different genital stages 1, 2, 3, 4, and 5 are different (*p* < 0.0001). There is no difference between genital stage 5 and adults (*p* > 0.05, <0.1). The small increase in volumes in genital stage 1 is thought to be related to proliferation of Sertoli cells. The increase in volume from genital stage 1 to 2, which indicates the beginning of puberty is related to the development of seminiferous tubules and the effect of FSH and is highly significant.

**Table 4:** Testicular volumes – (W-ss)^3^ X 0.88 by genital stages equivalent to ultrasound W x H x L × 0.71

Stage	Age in years range	# of testes	Volume in ml quartiles (25% to 75%)	Minimum to maximum ml
Stage 1				
1-a	3.4 to 6.5	24	0.50 to 0.71	0.50 to 0.71
1-b	7.2 to 8.7	24	0.71 to 0.77	0.60 to 0.96
1-c	8.9 to 13.2	32	0.71 to 1.27	0.60 to 1.27
Stage 1 All	3.4 to 13.2	80	0.71 to 0.96	0.50 to 1.27
Stage 2	10.0 to 15.0	34	2.68 to 4.00	2.16 to 6.52
Stage 3	11.4 to 15.0	22	5.48 to 6.42	4.64 to 7.47
Stage 4	11.7 to 16.9	44	8.27 to 12.32	6.13 to 12.32
Stage 5	13.0 to 17.5	38	15.47 to 17.32	10.71 to 21.46
Adult - All	15.8 to 34.0	100	15.29 to 21.24	10.57 to 21.24

Stage 1a, b, c: The only criterion for separation is age

## Discussion

The information presented would solve the problem of overestimation by orchidometers and reference to age, would permit assessment of the beginning and progression of puberty, of micro and macroorchidism, Klinefelter and other conditions mentioned. It would also give information on precocious development and late development (with some caution, because this is a cross sectional study and some of the subjects may have had that stage of genital development for some time). Nevertheless, the range of ages we observed are consistent with those reported by others [24, 25]. Longitudinal studies and additional number of subjects may provide more accurate information. Assessment of factors affecting differential testicular volumes of the left and right testis could be done by the formulas by the difference of the width obtained in the scrotum [16].

The penile measurements that we obtained are practically identical to those reported by Tomova in 6200 children (300 for every year of age) [26].

If we, presently, have a simple, low cost clinical method (a width of the testis obtained in the scrotum with a centimeter ruler) that closely matches the results obtained by ultrasound, this method would seem to be preferable. Orchidometers and calculation by the scrotal measurements mentioned, would not need to be used anymore.

Since we analyzed the overestimation of the US values by the orchidometers, it would appear appropriate to analyze the possible difference in ultrasound values for other measurements and those we are recommending.

Though the measurements of width, height, and length on ultrasound are done with an electronic caliper that can measure to within 0.1 mm, depending on the location and compression one can easily obtain a 1 mm variation. That is why it is usually recommended to obtain 3 measurements. Then, however, some use the highest measurements and others the average.

A 1 mm difference in the measurement of the width of the testis on ultrasound or in the scrotum will yield a difference of volume of 10% for adults (19.1 to 21.2 ml), 14% for pubertal (6.1 to 7.1 ml), or 26% for prepubertal (0.7 to 0.9 ml) males, variability that constitutes the normal range. A difference of the width of the left and right testes does not indicate an error in measurement. Of interest is that the measurement of the width in the scrotum seems quite accurate and statistically different for each genital stage (Table 2). Of the 318 testes we measured 93.6% had an equal width of the left and right testis, 2.8% had a 1 mm, 1.8% a 2 mm, and 1.8% a 3 mm difference.

A difference in the formulas of the H/W ratio used of 0.7 to 0.8 will yield a 14% difference for different cohorts in the US volumes, or 7% for 0.75 to 0.8 and so on. And a difference of the formulas of the L/W ratio of 1.5 to 1.55 will yield a difference of 7% in the US volumes. These differences do not indicate errors. Different cohorts maybe have different H/W or L/W ratios. It simply indicates that if one uses the same formula for all the cohorts the difference in volumes is minor.

The observations described should be helpful to assess the onset and progression and disorders of puberty and disorders previously mentioned. The US remains the method of choice for the evaluation of extra testicular (i.e. hydrocele, spermatocele, epididymal cyst, varicocele) or intratesticular (i.e. tumors) abnormalities.

The process for the determination of the testicular volume seems simple.

1. Measurement of the width of testis in the scrotum can be obtained by smoothing the scrotal skin around the testis, avoiding compression and using the ruler.
2. The genital stage of development is determined visually by the appearance of the penis testes and scrotum (Additional file 1: Figure S4), and the measurement of the width of the testis and, if needed, the penis (Table 2), without consideration of pubic hair.
3. The width is subtracted by the double scrotal skin for the genital stage. One could make it simpler by subtracting 1.5 mm for genital stages 1, 2, and 3 and 2 mm for genital 4, 5, and adults. The error or variation would be minor.
4. The volume, then, is calculated by formula (W-ss)^3^ × 0.88; and compared with the normal values for the genital stages and adults shown in Figs. 3 and 4.
5. If one would like to compare the values obtained by the formula with those obtained by ultrasound in the institution, one should use the formula equivalent to the ellipsoid equation that they use for the calculation of US volumes: for US equation W^2^ x L × 0.52 use formula (W-ss)^3^ × 0.8; for US equation W x H x L × 0.52 use formula (W-ss)^3^ × 0.64; and for US equation W x H x L × 0.71 use formula (W-ss)^3^ × 0.88.
6. To convert values calculated with (W-ss)^3^ × 0.88, with the constant of 0.71, to values obtained with the formula (W-ss)^3^ × 64 with the constant of 0.52, divide the values by 1.365 (0.71/0.52 = 1.365) or vice versa.

We certainly encourage the readers to use the formulas (or to develop formulas) and compare the volumes with those obtained on US in their institution, to confirm that volumes matching US volumes can be obtained with a simple measure of the width of the testis in the scrotum. More observations could provide information on accuracy and inter-observant difference.

## Conclusion

With a simple measurement of the width of the testis in the scrotum, with a centimeter ruler, testicular volumes matching US values were calculated and normative values for each stage of genital development were determined. This information should solve the present problems of overestimation of US values with orchidometers or external linear measurements, and the lack of normative values for stages of genital development.

## Additional file

Additional file 1: Figure S1. Testicular volumes determined by the Prader orchidometer (Goede et al.) [8] and in our subjects by measurements in the scrotum with a centimeter ruler (W^2^ x L × 0.52). **Figure S2**. Testicular volumes for different stages of genital development (G), obtained with the formula (w-ss)3 × 88, equivalent to ultrasound W x H x L × 0.71. The boxes show the volumes in ml in the ordinance with the range of ages in the abscissa. Additional file 1: **Figure S3**. Testicular volumes for different Genital Stages obtained by measurements in the scrotum with a centimeter ruler (W^2^ x L × 0.52). The boxes show the volumes in ml in the ordinance with the range of ages in the abscissa. (The volumes are similar to those obtained with a Prader orchidometer by Goede et al. (Additional file 1: Figure S1).) **Figure S4**. A rough drawing of a photograph published in 1971, for determining stages of genital development (G), (testes, scrotum and penis only), without consideration of pubic hair from Van Wieringen JD, Wafelbakker F, Verbrugge HP, et al. Growth Diagrams 1965 Netherlands: Second National Survey on 0–24 Year Olds. Netherlands Institute for Preventative Medicine TNO. Groningen, The Netherlands: Wolters- Noordhoff; 1971. **Table S1**. Similarity of Our Volumes to those Obtained by US (Normalized Smoothed) Reported by Joustra et al. [22].

## Abbreviations

FSH: Follicle Stimulating Hormone
H: Height
IGSF1: Immunoglobulin super family member 1
IRB: Institutional Review Board
L: Length
LH: Luteinizing Hormone
SD: Standard Deviation
ss: scrotal skin
US: ultrasound
W: Width
W-ss: Width minus scrotal skin
π: pi
