# Erratum to: Appraisal of testicular volumes: volumes matching ultrasound values referenced to stages of genital development

**DOI:** 10.1186/s13633-017-0050-1

**Published:** 2017-09-29

**Authors:** Juan F. Sotos, Naomi J. Tokar

**Affiliations:** 1 Department of Pediatric, College of Medicine, The Ohio State University, Nationwide Children’s Hospital, Section of Pediatric Endocrinology, 700 Children’s Drive, Columbus, OH 43205 USA; 2 Section of Pediatric Endocrinology, Nationwide Children’s Hospital, 700 Children’s Drive, Columbus, OH 43205 USA

## Correction

The original article shows some incorrect values in Table 1 of the Supplement. The table shown below is as it should be. The volumes at age 4, 6 & 8 have been changed from “0.5 to 9.0” (W-ss)^3^ x 0.64 in ml, to “0.5 to 0.9”.

**Table 1:** Similarity of Our Volumes to those Obtained by US (Normalized Smoothed) Reported by Joustra et al. [22]

Age	(W-ss)^3^ x 0.64 in ml in Figure 2 of manuscript	Range in ml [22] (W x H x L x 0.52) Mean ± 2 SD
4	0.5 to 0.9	0.3 to 0.85
6	0.5 to 0.9	0.3 to 1.1
8	0.5 to 0.9	0.3 to 1.2
13	0.5 to 9.0	0.75 to 11.0
14	1.5 to 15.5	1.5 to 14.5
15	1.5 to 15.7	3.0 to 16.0
16	4.5 to15.7	4.5 to 18.0
17	7.7 to 15.7	6.3 to 19.0
Adult	7.7 to 15.7	7.5 to 20.0
