# Supplementary material for: Appraisal of testicular volumes: volumes matching ultrasound values referenced to stages of genital development
Source: Int J Pediatr Endocrinol. 2017 Jul 17;2017:7. doi: 10.1186/s13633-017-0046-x (PMC5513322; doi:10.1186/s13633-017-0046-x)
Supplement: Additional file 1: Figure S1. — Testicular volumes determined by the Prader orchidometer (Goede et al.) [8] and in our subjects by measurements in the scrotum with a centimeter ruler (W2 x L × 0.52). Figure S2. Testicular volumes for different stages of genital development (G), obtained with the formula (w-ss)3 × 88, equivalent to ultrasound W x H x L × 0.71. The boxes show the volumes in ml in the ordinance with the range of ages in the abscissa. Additional file 1: Figure S3. Testicular volumes for different Genital Stages obtained by measurements in the scrotum with a centimeter ruler (W2 x L × 0.52). The boxes show the volumes in ml in the ordinance with the range of ages in the abscissa. (The volumes are similar to those obtained with a Prader orchidometer by Goede et al. (Additional file 1: Figure S1).) Figure S4. A rough drawing of a photograph published in 1971, for determining stages of genital development (G), (testes, scrotum and penis only), without consideration of pubic hair from Van Wieringen JD, Wafelbakker F, Verbrugge HP, et al. Growth Diagrams 1965 Netherlands: Second National Survey on 0–24 Year Olds. Netherlands Institute for Preventative Medicine TNO. Groningen, The Netherlands: Wolters- Noordhoff; 1971. Table S1. Similarity of Our Volumes to those Obtained by US (Normalized Smoothed) Reported by Joustra et al. [22]. [file 13633_2017_46_MOESM1_ESM.docx]

**Additional File (Supplement)**

**Ellipsoid Equations & Formulas**

There are a number of ellipsoid shapes and equations to determine their volumes.

Terms: W = width; H = Height, depth, or thickness; L = length; $\left( \frac{\boldsymbol{W}}{\boldsymbol{2}} \right)$ = radius (r); $\left( \frac{\boldsymbol{W}}{\boldsymbol{2}} \right)^{\boldsymbol{2}}$x $\boldsymbol{\pi}$ = area of circle.

For a **Cylinder** the calculation of the volume is simple:

$$\left( \frac{\boldsymbol{W}}{\boldsymbol{2}} \right)^{\boldsymbol{2}}\boldsymbol{\times\pi}$$

***r_1_***

***r_2_***

$$\boldsymbol{\times L}$$

**`````**

$\boldsymbol{=}$ $\boldsymbol{Volume}$

$\left( \frac{\boldsymbol{W}}{\boldsymbol{2}} \right)^{\boldsymbol{2}}\boldsymbol{\times\pi\times L}$=

$$\left( \frac{W}{2} \right)^{2}\times\pi\times L=Volume$$

The ellipsoids have a different shape than a cylinder.

The **Prolate Ellipsoid** (Prolate spheroid) have the shape of a rugby ball so the volume would be the area of the circle times the different length:

***r_3_***

$$\boldsymbol{\times}\frac{\boldsymbol{4}}{\boldsymbol{3}}\boldsymbol{\times}\frac{\boldsymbol{L}}{\boldsymbol{2}}$$

***r_1_***

***r_2_***

$$\left( \frac{\boldsymbol{W}}{\boldsymbol{2}} \right)^{\boldsymbol{2}}\boldsymbol{\times\pi}$$

When resolved would be:

$$W^{2}\times L\times0.52=Volume$$

$\boldsymbol{W}^{\boldsymbol{2}}\boldsymbol{\times L\times}\frac{\boldsymbol{\pi}}{\boldsymbol{6}}$ =

The beads on the Prader orchidometer are prolate ellipsoids.

**Rotational Ellipsoid**: The circle is somewhat flattened, so the Height is less than the Width.

$$\boldsymbol{\times}\frac{\boldsymbol{4}}{\boldsymbol{3}} \boldsymbol{\times}\frac{\boldsymbol{L}}{\boldsymbol{2}}$$

***r_1_***

***r_2_***

$$\frac{\boldsymbol{W}}{\boldsymbol{2}}\boldsymbol{\times}\frac{\boldsymbol{H}}{\boldsymbol{2}}\boldsymbol{\times\pi}$$

***r_3_***

When resolved would be:

$$\boldsymbol{W\times H\times L \times}\frac{\boldsymbol{\pi}}{\boldsymbol{6}}\boldsymbol{=}$$

$$W\times H\times L\times0.52=Volume$$

This is the equation used in ultrasonography since the Height is usually measured.

**Rotational Ellipsoid** equation when the constant of 0.71 (Suggested by Lambert [13]) is used to closely match the true testicular volumes obtained by water displacement.

$$W\times H\times L\times0.71=Volume$$

**Formulas:**

Formulas equivalent to the ellipsoid equations used, with inclusion of the values observed in ultrasound measurements, were developed to approximate or match ultrasound volumes, with correction of the width and length of the testes obtained in the scrotum, to avoid inclusion of the scrotal skin and epididymis.

Terms: W-ss = width minus double scrotal skin; Height expressed as the $\frac{H}{W}$ ratio of the width observed in our hospital US, (W-ss x $\frac{H}{W}$ = W-ss x 0.8); and the Length expressed as the $\frac{L}{W}$ ratio of the width observed in US in our hospital (W-ss) x $\frac{L}{W}$ = (W-ss) x 1.55.

For the Equation W^2^ X L X .52, the equivalent formula would be (W-ss)^2^ x (W-ss x 1.55) x 0.52 = (W-ss)^3^ x 0.8)

For the US equation: W x H x L x $\frac{}{6}$ equal to W x H x L x 0.52, the equivalent formula would be (W-ss) x (W-ss x 0.8) x (W-ss x 1.55) x 0.52 = (W-ss)^3^ x 0.64.

If the constant 0.71 instead of 0.52 is used, then the US equation would be W x H x L x 0.71, and the equivalent formula W-ss x (W-ss x 0.8) x (W-ss x 1.55) x 0.71 = (W-ss)^3^ x 0.88.

The double scrotal skin measured 1.7 mm for genital development stage one (G1), 1.5 mm for G2, 1.6 mm for G3, 1.9 mm for G4, 2.0 mm for G5, and 2.1 mm for adults [16].

The length (height or depth) of the head of the epididymis by ultrasound in mm [5] is: 4.5 to 6.0 for prepubertal 2 to 10 years; 6.5 to 8.0 for pubertal 11 to 14 years; and 9.5 to 11.4 for pubertal 15 to 17 years.

**Legends**

Supplement Figure 1. Testicular volumes determined by the Prader orchidometer (Goede *et al.*)[8] and in our subjects by measurements in the scrotum with a centimeter ruler (W^2^ x L x 0.52).

Supplement Figure 2. Testicular volumes for different stages of genital development (G), obtained with the formula (w-ss)3 x 88, equivalent to ultrasound W x H x L x 0.71. The boxes show the volumes in ml in the ordinance with the range of ages in the abscissa.

Supplement Figure 3. Testicular volumes for different Genital Stages obtained by measurements in the scrotum with a centimeter ruler (W^2^ x L x 0.52). The boxes show the volumes in ml in the ordinance with the range of ages in the abscissa. (The volumes are similar to those obtained with a Prader orchidometer by Goede *et al.* (Supplement Figure 1).)

Supplement Figure 4. A rough drawing of a photograph published in 1971, for determining stages of genital development (G), (testes, scrotum and penis only), without consideration of pubic hair from Van Wieringen JD, Wafelbakker F, Verbrugge HP, *et al*. Growth Diagrams 1965 Netherlands: Second National Survey on 0-24 Year Olds. Netherlands Institute for Preventative Medicine TNO. Groningen, The Netherlands: Wolters- Noordhoff; 1971.

| **Similarity of Our Volumes to those Obtained**  **by US (Normalized Smoothed) Reported by Joustra *et al.* [22]** | | |
| --- | --- | --- |
| Age | (W-ss)^3^ x 0.64 in ml  in Figure 2 of manuscript | Range in ml [22]  (W x H x L x 0.52)  Mean ± 2 SD |
| 4 | 0.5 to 9.0 | 0.3 to 0.85 |
| 6 | 0.5 to 9.0 | 0.3 to 1.1 |
| 8 | 0.5 to 9.0 | 0.3 to 1.2 |
| 13 | 0.5 to 9.0 | 0.75 to 11.0 |
| 14 | 1.5 to 15.5 | 1.5 to 14.5 |
| 15 | 1.5 to 15.7 | 3.0 to 16.0 |
| 16 | 4.5 to15.7 | 4.5 to 18.0 |
| 17 | 7.7 to 15.7 | 6.3 to 19.0 |
| Adult | 7.7 to 15.7 | 7.5 to 20.0 |

Supplement Table 1

**JF Sotos**


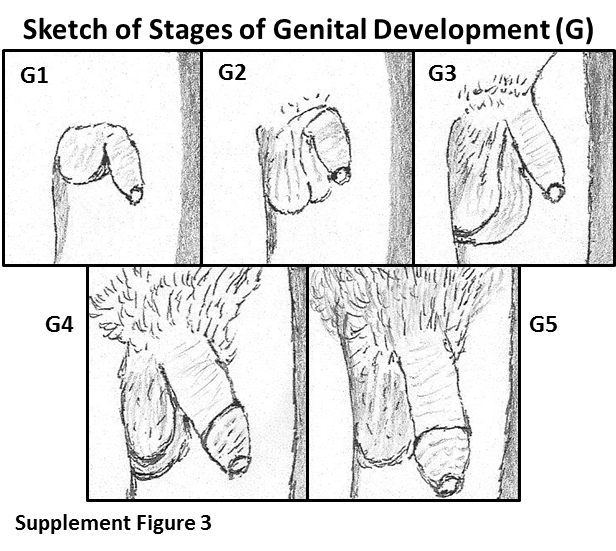


**Supplement Figure 4:** The main characteristics of the stages of genital development (testis, scrotum and penis), independent of pubic hair development, were well defined by Tanner [19]: G1. Prepubertal or preadolescent; the testes and the scrotum are small and penis is as in early childhood. G2. The testes and the scrotum have enlarged and there is some reddening of the scrotal skin. The penis is still as in early childhood. G3. Growth of the penis in both length and breadth and further growth of testes and the scrotum. G4. The penis has further enlarged in length and breadth with development of the glans. The testes and scrotum are further enlarged. G5. Genitalia are adult in size and shape.
